# GlycA, a novel marker for low grade inflammation, reflects gut microbiome diversity and is more accurate than high sensitive CRP in reflecting metabolomic profile

**DOI:** 10.1007/s11306-020-01695-x

**Published:** 2020-06-20

**Authors:** Kati Mokkala, Noora Houttu, Ella Koivuniemi, Nikolaj Sørensen, Henrik Bjørn Nielsen, Kirsi Laitinen

**Affiliations:** 1 Institute of Biomedicine, Research Centre for Integrative Physiology and Pharmacology, University of Turku, 20014 Turku, Finland; 2 Clinical Microbiomics, Copenhagen, Denmark; 3 Department of Obstetrics and Gynecology, Turku University Hospital, 20521 Turku, Finland

**Keywords:** Low grade inflammation, GlycA, hsCRP, Metabolomics, Gut microbiome diversity

## Abstract

**Introduction:**

Gut microbiota is, along with adipose tissue, recognized as a source for many metabolic and inflammatory disturbances that may contribute to the individual’s state of health.

**Objectives:**

We investigated in cross-sectional setting the feasibility of utilizing GlycA, a novel low grade inflammatory marker, and traditional low grade inflammatory marker, high sensitivity CRP (hsCRP), in reflecting serum metabolomics status and gut microbiome diversity.

**Methods:**

Fasting serum samples of overweight/obese pregnant women (n = 335, gestational weeks: mean 13.8) were analysed for hsCRP by immunoassay, GlycA and metabolomics status by NMR metabolomics and faecal samples for gut microbiome diversity by metagenomics. The benefits of GlycA as a metabolic marker were investigated against hsCRP.

**Results:**

The GlycA concentration correlated with more of the metabolomics markers (144 out of 157), than hsCRP (55 out of 157) (FDR < 0.05). The results remained essentially the same when potential confounding factors known to associate with GlycA and hsCRP levels were taken into account (P < 0.05). This was attributable to the detected correlations between GlycA and the constituents and concentrations of several sized VLDL-particles and branched chain amino acids, which were statistically non-significant with regard to hsCRP. GlycA, but not hsCRP, correlated inversely with gut microbiome diversity.

**Conclusion:**

GlycA is a superior marker than hsCRP in assessing the metabolomic profile and gut microbiome diversity. It is proposed that GlycA may act as a novel marker that reflects both the gut microbiome and adipose tissue originated metabolic aberrations; this proposal will need to be verified with regard to clinical outcomes.

**Clinical trial registration:**

ClinicalTrials.gov, NCT01922791, August 14, 2013

**Electronic supplementary material:**

The online version of this article (10.1007/s11306-020-01695-x) contains supplementary material, which is available to authorized users.

## Introduction

Low grade inflammation is a condition characterized by increased concentrations of serum inflammatory markers and it is associated with many metabolic disturbances like insulin resistance (Minihane et al. 2015). It is typically detected in obese individuals and has been linked with diseases such as type 2 diabetes and dyslipidemia (Jung and Choi 2014). At present, the presence of low grade inflammation is typically evaluated by increased concentrations of circulating high sensitive C-reactive protein CRP (hsCRP), a marker which is an early single acute phase protein produced in the liver as a response to the cytokine, IL-6 (Pearson et al. 2003, Sproston and Ashworth 2018).

GlycA is a novel marker of low grade inflammation; it reflects the glycosylation of acute phase proteins (Bell et al. 1987; Otvos et al 2015). Similar to hsCRP, the concentration of GlycA has been related to multiple metabolic aberrations including type 2 diabetes and cardiovascular diseases (Connelly et al. 2017). As GlycA consists of a complex heterogeneous signal, it has been proposed to reflect better than hsCRP the systemic acute phase response (Otvos et al. 2015; Ritchie et al. 2015, 2019).

One of the well established sources for low grade inflammation is adipose tissue (Calder et al. 2011). Recently, the gut microbiota, i.e. the composition and the metabolites produced by bacteria, has emerged as a significant contributor to low grade inflammation (Tilg et al. 2019). Changes in gut microbiota, e.g. lower gut microbiome richness, have been found in association with low grade inflammation, insulin resistance and dyslipidemia (Le Chatelier et al. 2013). In our previous study with overweight and obese pregnanct women, we detected an inverse association between GlycA and gut microbiota richness, analysed using 16S rRNA sequencing (Röytiö et al. 2017). Low grade inflammation may originate from the lower amount of bacteria supporting the intestinal barrier integrity (Tilg et al. 2019). Subsequently, lipopolysaccharide, LPS, may enter circulation and induce metabolic endotoxemia, i.e. a doubling or even trebling of the levels of highly antigenic LPS in the circulation (Cani et al. 2007).

The aim of this study was to investigate the extent to which GlycA and hsCRP reflect host metabolomics, analysed by NMR-approach and gut microbiome diversity, as determined by metagenomic analyses.

## Subjects and methods

### Participants and design

In this cross-sectional study, markers for low grade inflammation and metabolomic profiles were analysed from fasting serum samples of women participating in a mother-infant dietary intervention trial being conducted in southwest Finland. This single-center trial was executed in the Turku University Hospital and University of Turku in Finland with recruitment between October 2013 and July 2017 (ClinicalTrials.gov, NCT01922791). Details of research design and methods have been previously described (Pellonperä et al. 2019). Of the 439 recruited women, 335 women at the baseline (i.e. prior to intervention onset, gestational weeks mean 13.8) were included in this study following the exclusion of women with self-reported acute infections, those who had used antibiotics within two weeks before and after the study visit to exclude those individuals who may have a nonsymptomatic inflammation and those who had hsCRP ≥ 10 mg/l, indicative of a possible infection.

The characteristics of the women (Table 1), including age, education and smoking were collected from questionnaires. Prepregnancy BMI (kg/m^2^) was calculated by dividing self-reported weight in kilograms, obtained from women’s welfare clinic records, by height measured with a wall stadiometer to the nearest 0.1 cm at the first study visit. Dietary intake of fibre was calculated from three-day-food diaries recorded within a week prior to study visit.

**Table 1:** Clinical and inflammatory characteristics of the women

	Mean (SD)
Clinical characteristics	
Age (years)	30.9 (4.6)
Prepregnancy BMI (kg/m^2^)	29.14 (3.81)
Obese, n (%)	117/335 (35%)
Highly educated, n (%)	192/300 (64%)
Primipara, n (%)	164/335 (49%)
Smoking before pregnancy, n (%)	73/302 (24%)
Smoking at early pregnancy, n (%)	17/301 (5.6%)
Gestational weeks at the study visit	13.8 (2.2)
Inflammatory and metabolomic markers	
hsCRP (mg/l), n = 335	4.69 (2.43)
GlycA (mmol/l), n = 335	1.19 (0.10)
LPS activity (EU/ml), n = 146	0.154 (0.04)
Gut microbiome metagenomic diversity	
Shannon gene diversity index, n = 321	16.71 (0.73)
Gene-richness, n = 321	465,556.21 (107,818.11)
Shannon species diversity index, n = 321	2.67 (0.31)
Species richness, n = 321	242.64 (58.44)

### Metabolomics

Fasting (10 h minimum) blood samples were drawn from the antecubital vein at early pregnancy (mean gestational weeks 13.8), and the serum was separated and frozen in aliquots at − 80 °C until being analyzed for serum metabolomics. A high-throughput proton NMR metabolomics platform (Nightingale, Helsinki, Finland) was used to analyze the serum metabolic profile as described earlier (Soininen et al. 2015). The analysis platform assesses 228 variables, including biomarkers of lipid and glucose metabolism, amino acids, ketone bodies and GlycA. GlycA consists of a complex heterogeneous nuclear magnetic resonance signal originating from the N-acetyl sugar groups present on multiple acute phase glycoproteins in the circulation; α1-acid glycoprotein, haptoglobin, α1-antitrypsin, α1-antichymotrypsin and transferrin (Bell et al. 1987; Otvos et al. 2015).

### Gut microbiome diversity

Fecal samples were collected in sterile plastic pots on the morning of the study visit at early pregnancy (mean gestational weeks 13.8), or on the previous evening, delivered to the study unit and kept at − 20 °C until DNA extraction. DNA was extracted from 50 mg of homogenized feces using a GTX stool extraction kit and a fully automated GenoXTract machine (Hain Lifescience, Nehren, Germany) as previously described (Mokkala et al. 2016). Prior to extraction, mechanical lysis was performed by bead-beating the samples in ceramic bead tubes with MOBIO PowerLyzerTM 24 Bench Top Bead-Based Homogenizer (MO BIO Laboratories, Inc., USA). The DNA concentrations were measured with Qubit 2.0 dsDNA HS assay kit (Life Technologies), after which the DNAs were stored at − 80 °C until sequencing.

The genomic DNA was randomly sheared into fragments of approximately 350 bp. The fragmented DNA was used for library construction with NEBNext Ultra II Library Prep Kit for Illumina (New England Biolabs). The prepared DNA libraries were evaluated using Qubit 2.0 fluorometric quantitation and an Agilent 2100 Bioanalyzer for the fragment size distribution. Quantitative real-time PCR (qPCR) was used to determine the concentration of the final library before sequencing. The library was sequenced using 2 × 150 bp paired-end sequencing on an Illumina HiSeq platform. The raw FASTQ files were quality controlled using KneadData (v. 0.6.1) to remove low-quality bases and reads derived from the host genome as follows: Using Trimmomatic (v. 0.36), the reads were quality trimmed by removing Nextera adapters, leading or trailing bases with a Phred score below 20, and trailing bases in which the Phred score over a window of size 4 drops below 20. Trimmed reads shorter than 100 bases were discarded. Reads that mapped to the human reference genome GRCh38 (with Bowtie2 v. 0.2.3.2 using default settings) were also discarded. Read pairs in which both reads passed filtering were retained; these were classified as high quality non-host (HQNH) reads. HQNH reads were mapped to the integrated gene catalog (IGC) (Li et al. 2014) using BWA mem (v. 0.7.16a) with options to increase accuracy (-r 1 -D 0.3). PCR/optical duplicates were removed using samtools (v. 1.6). A read pair where both reads had a mapping quality (MAPQ) ≥ 20 and an alignment of at least 100 bp and with ≥ 95% identity to a single IGC gene was considered mapped. However, the mapping was rejected if > 10 bases at either end of the read failed to align to an existing gene sequence (i.e. alignment beyond the IGC gene sequence was accepted). The read counts were used to estimate the abundance of the Clinical Microbiomics proprietary set of IGC metagenomic species (MGS) (Nielsen et al. 2014) derived from abundance profiles across 3200 reference samples. For each MGS, we defined the “core” genes as the 100 genes with the highest correlation of abundance across the reference samples. A table of MGS counts was created based on the total gene counts for the 100 core genes of each MGS. However, an MGS was considered as detected only if the read pairs were mapped to at least three of the 100 core genes; MGSs that did not satisfy this criterion were designated as zero counts. The relative abundance estimate of each MGS was made by normalizing the counts for gene lengths. Rarefied (downsampled) MGS abundance profiles were calculated by performing the above procedure on a rarefied gene counts table (generated by random sampling, without replacement, of HQNH read pairs).

The gene and species richness and the Shannon diversity index of samples were calculated (vegan R package) (Oksanen et al. 2017) from the number of genes or MGSs that were detected and their relative abundances in the downsampled (7,281,907 read pairs) data. Richness describes the number of species (or genes) that is detected in a sample, whereas the Shannon diversity index also takes the relative abundance of the species (or genes) into account. A community dominated by a few abundant species (even though the total number of species may be high) will have a relative low diversity score, whereas communities with many similarly abundant species will have a higher diversity. Moreover, diversity is less sensitive to sampling errors, as it confers a higher weight to the more abundant species than to their rarer counterparts.

LPS activity, a marker of metabolic endotoxemia, was analyzed from serum samples (University of Helsinki, Finland) using a Limulus amebocyte lysate assay coupled with a chromogenic substrate (HyCult Biochemistry B.V., Uden, the Netherlands).

High-sensitivity C-reactive protein (hsCRP) was determined by using an automated colorimetric immunoassay on the Dade Behring Dimension RXL autoanalyzer (Siemens Healthcare, Camberly, Surrey, UK).

This study was conducted according to the guidelines laid down in the Declaration of Helsinki as revised in 2013, and all procedures that involved human subjects were approved by the Ethics Committee of the Hospital District of Southwest Finland (permission number 115/180/2012) and all participants provided written informed consent.

### Statistics

Pearson correlation was used to investigate the extent to which GlycA and CRP reflect metabolomic and gut microbiome diversity. In this calculation, those variables which were not normally distributed (skewness > 1), were natural-logarithmic transformed. The P-values were adjusted for multiple correction using the Benjamini–Hochberg (BH)- procedure (false discovery rate (FDR < 0.05 considered significant). In the analysis of the contribution of both inflammatory markers on metabolic variables, a multivariate linear model was devised with prepregnancy BMI and serum triglycerides as a counfounding factors. Further, we also added gestational weeks to the model to take into account the possible impact of pregnancy duration on the levels of GlycA and hsCRP. The inclusion of serum triglycerides in the model is based on the technical aspect of the NMR, i.e. the NMR-signal of GlycA may include signals from proteins and VLDL particles that carry the bulk of circulating triglycerides (TG) (Connelley et al. 2017) (personal communication from Nightingale). In the comparison of the unstandardized β-value between hsCRP and GlycA, the metabolomics variables in multivariable linear model, hsCRP, GlycA, triglycerides, prepregnancy BMI and gestational weeks, were divided by their standard deviation. When investigating the association between inflammatory markers and gut microbiome diversity, intake of fibre was included in the multivariate linear model as a possible confounding factor, as in previous study, fibre correlated with gut microbiota richness (Röytiö et al. 2017). The multiple correction was not performed to the P-values obtained from multiple linear regression due to the fact that our aim was to compare the association of the inflammatory marker with individual metabolomic markers.

## Results

### Characteristics of the women

The women were overweight (65%) or obese (35%), the mean prepregnancy BMI of all women being 29.1 (SD 3.8) (Table 1). Over half of the women were highly educated with a college or university degree. The concentration of hsCRP and GlycA, activity of LPS and the indexes of gut microbiome diversity are presented in Table 1. GlycA and hsCRP correlated with prepregnancy BMI (GlycA, r = 0.275, P < 0.001, hsCRP r = 0.269, P < 0.001, respectively). As expected, there was a clear correlation between the values of GlycA and hsCRP (r = 0.310, P < 0.001).

### GlycA and hsCRP differentially reflect the serum metabolomics

**Fig. 1:**
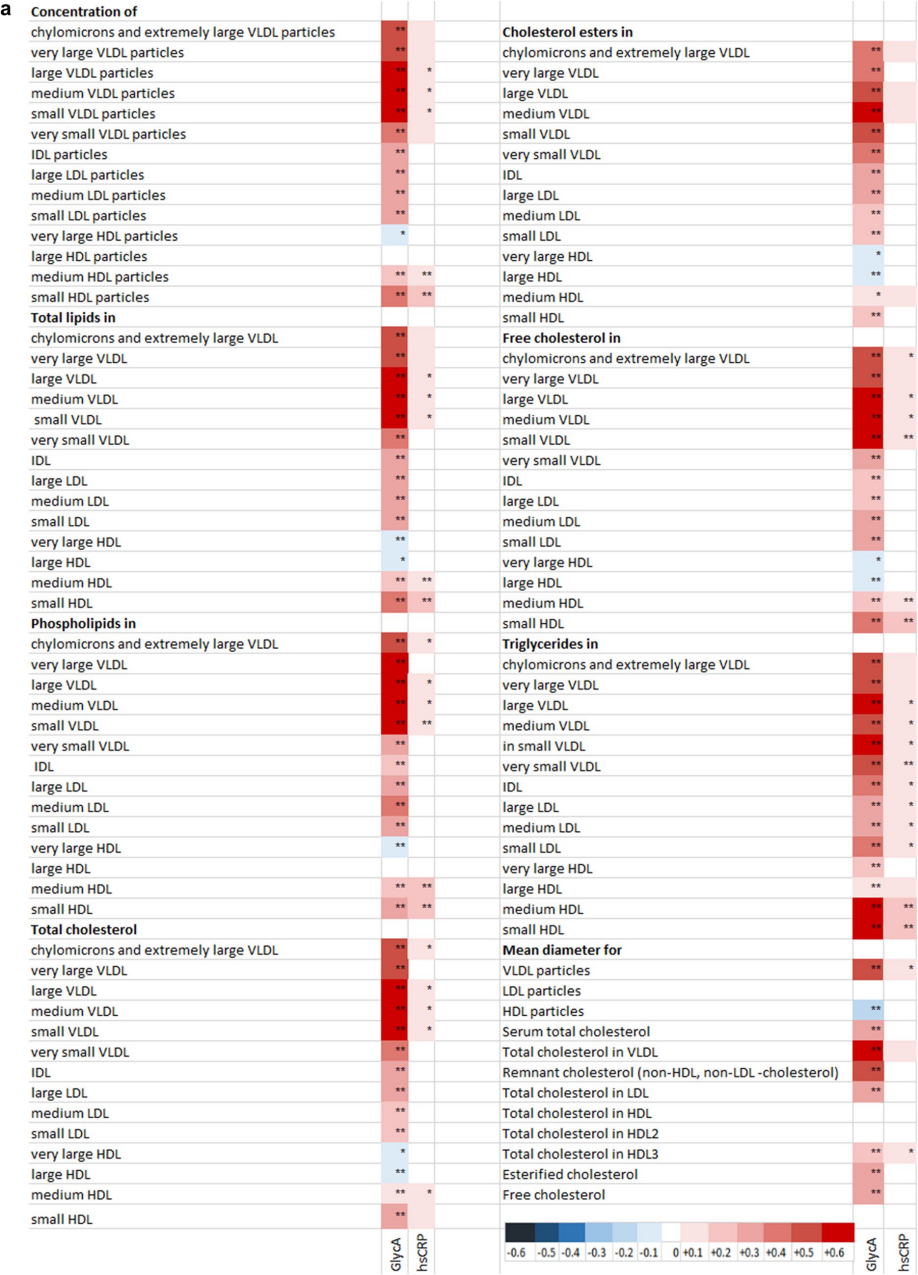

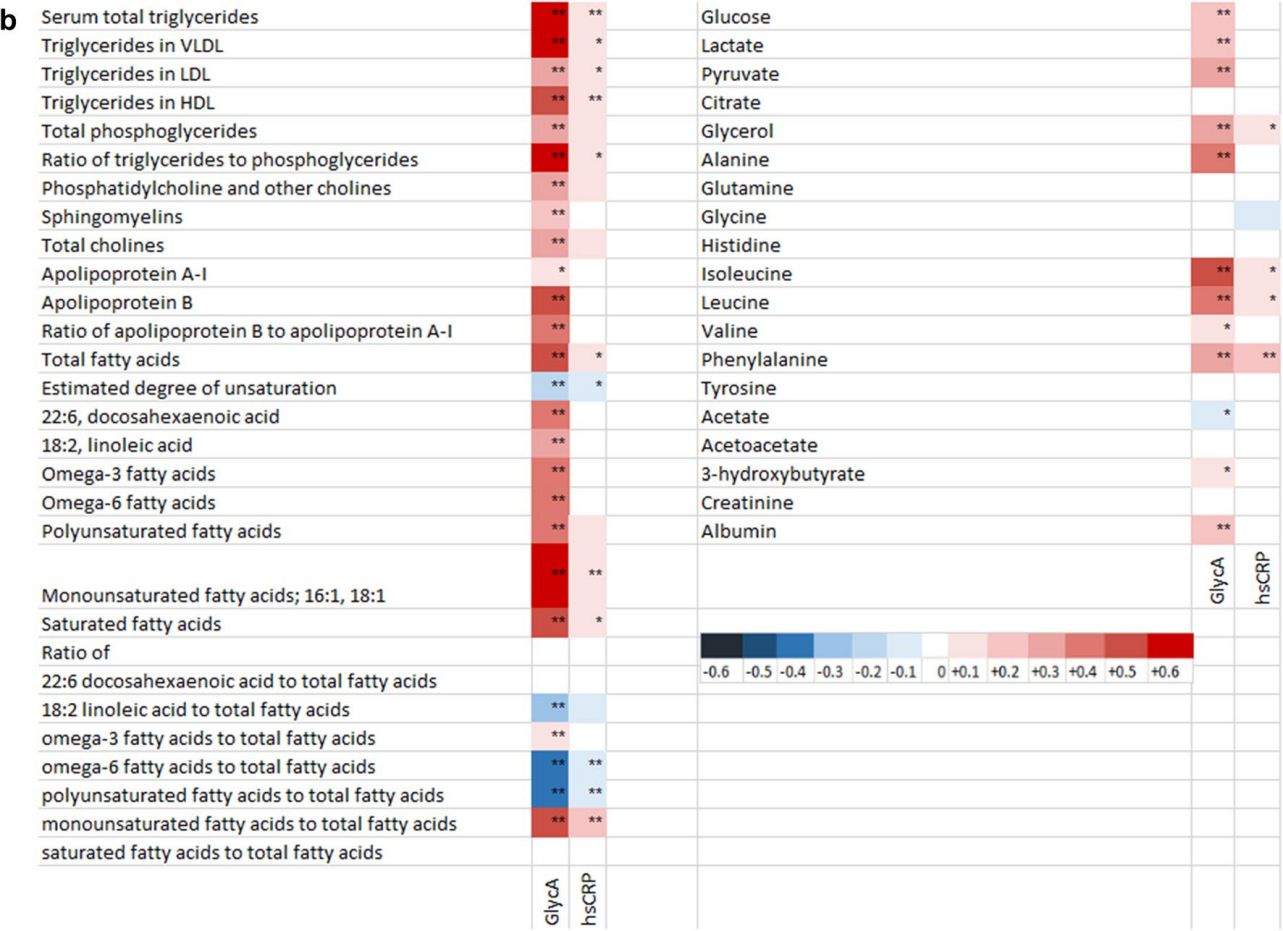
Heatmap of the Pearson correlation between inflammatory markers and metabolomics variables. *FDR < 0.05; **FDR < 0.01

GlycA was more sensitive than hsCRP in reflecting the metabolic status of the participants. This was indicated by the fact that a larger number of the metabolomics markers, 144 out of 157, correlated with GlycA, while hsCRP correlated with 55 out of 157 markers (heatmap Fig. 1). When concentrating on the lipid metabolites, both markers correlated with the concentration of several sized VLDL-particles and medium and small sized HDL-particles and their constituents. In addition to having higher regression coefficients values for all of these lipoproteins, the GlycA concentration also correlated with other lipoprotein particles and their constituents, including positive correlations with the concentrations of LDL-, IDL-particles and inverse correlations with very large and large HDL- particles and their constituents, while hsCRP correlated only with the levels of triglycerides of LDL and IDL-particles. When amino acids were examined separately, the concentration of GlycA was shown to correlate with the amounts of branched chain amino acids isoleucine, leucine and valine, and phenylalanine, while hsCRP correlated only with those isoleucine, leucine and phenylalanine, and even then, with lower correlation coefficient values.

### Lower gut microbiome diversity and serum LPS activity is related to higher GlycA

When the correlations with gut microbiome diversity were assessed, the level of GlycA reflected gut microbiome diversity (Gene Shannon, r = − 0.130, P = 0.020; Gene Richness, r = − 0.171, P = 0.002; MGS Shannon, r = − 0.134, P = 0.016; MGS Richness, r = − 0.196, P < 0.001), which, in contrast, displayed no correlation with hsCRP (P > 0.329 for all indexes). In a subset of samples (n = 146), we investigated the correlation between serum LPS and the inflammatory markers and observed a direct correlation between the levels of GlycA and LPS (r = 0.43, P < 0.001), but again, there was no evidence of any correlation between hsCRP and LPS (r = 0.079, P = 0. 346).

### GlycA reflect metabolic status independently of hsCRP

As GlycA was more sensitive at reflecting the metabolomic and gut microbiome diversity and further as hsCRP and GlycA correlated, we investigated whether the relationship between metabolomics markers and GlycA would be independent of hsCRP. Therefore, we devised a multilinear regression model in which both GlycA and hsCRP were included as dependent variables with prepregnancy BMI, serum triglycerides and gestational weeks as confounding factors.

**Fig. 2:**
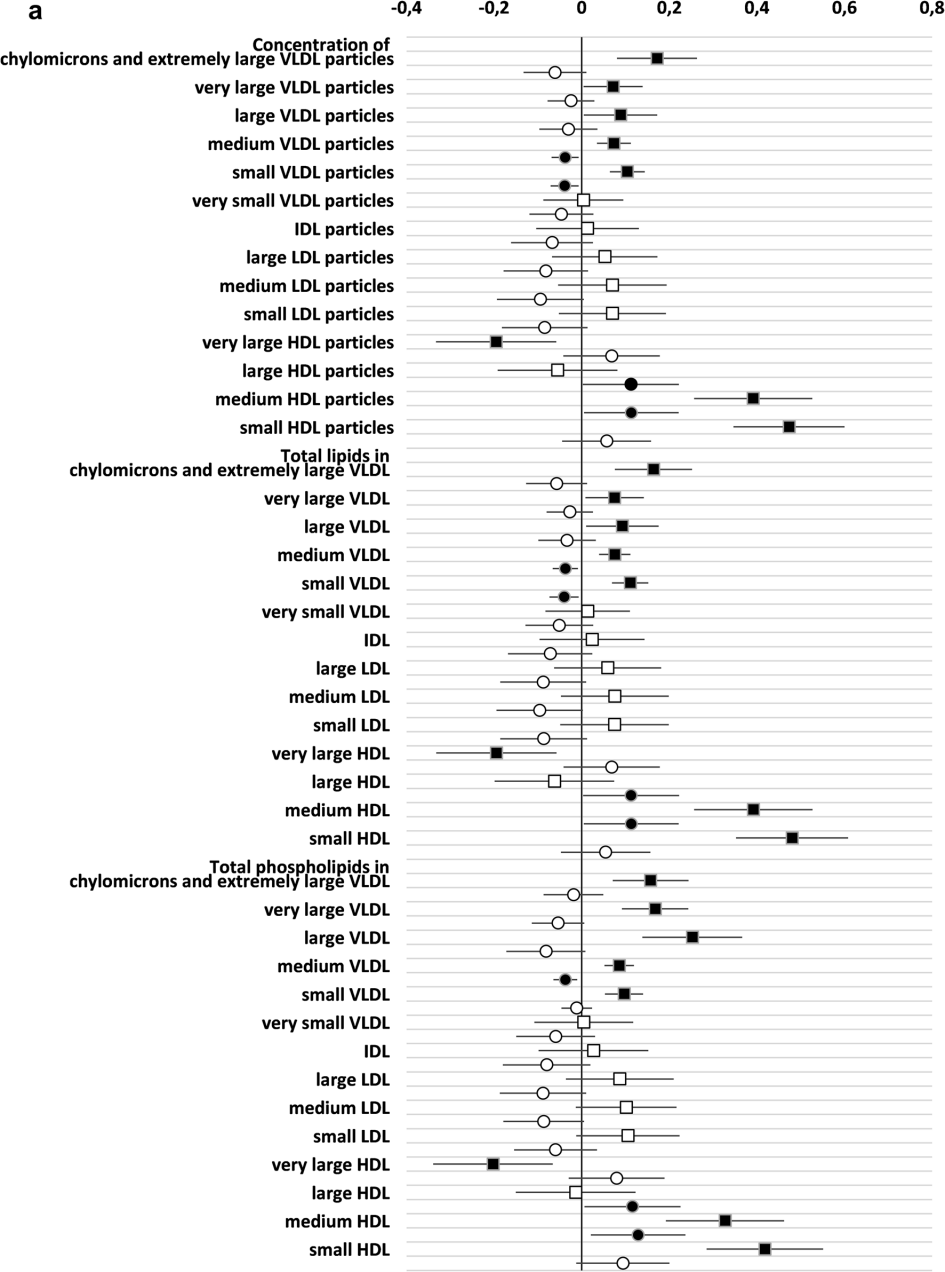

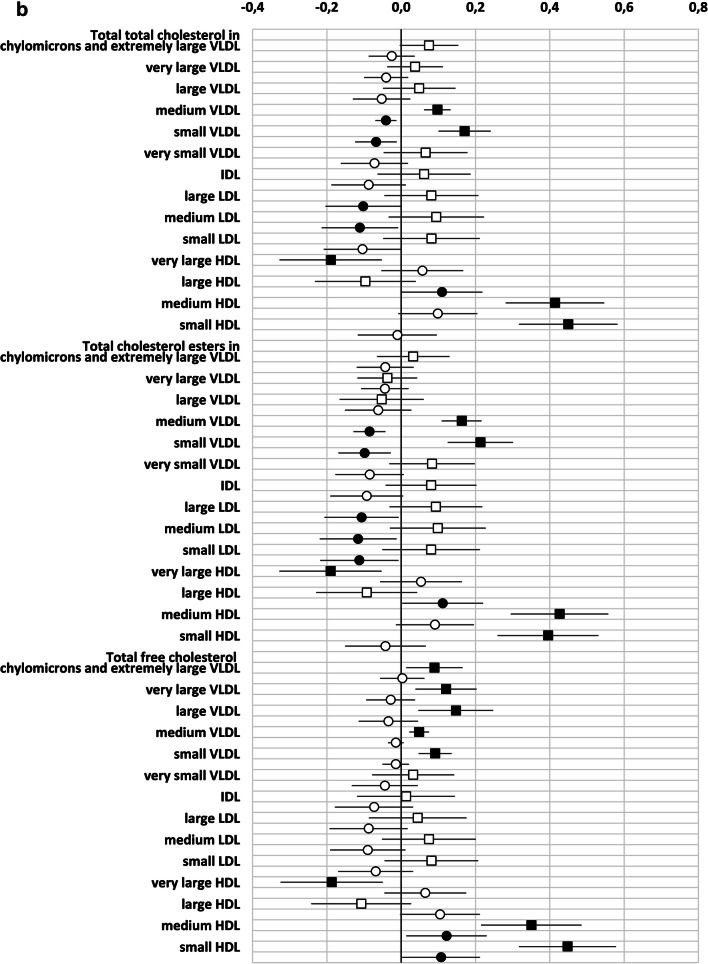

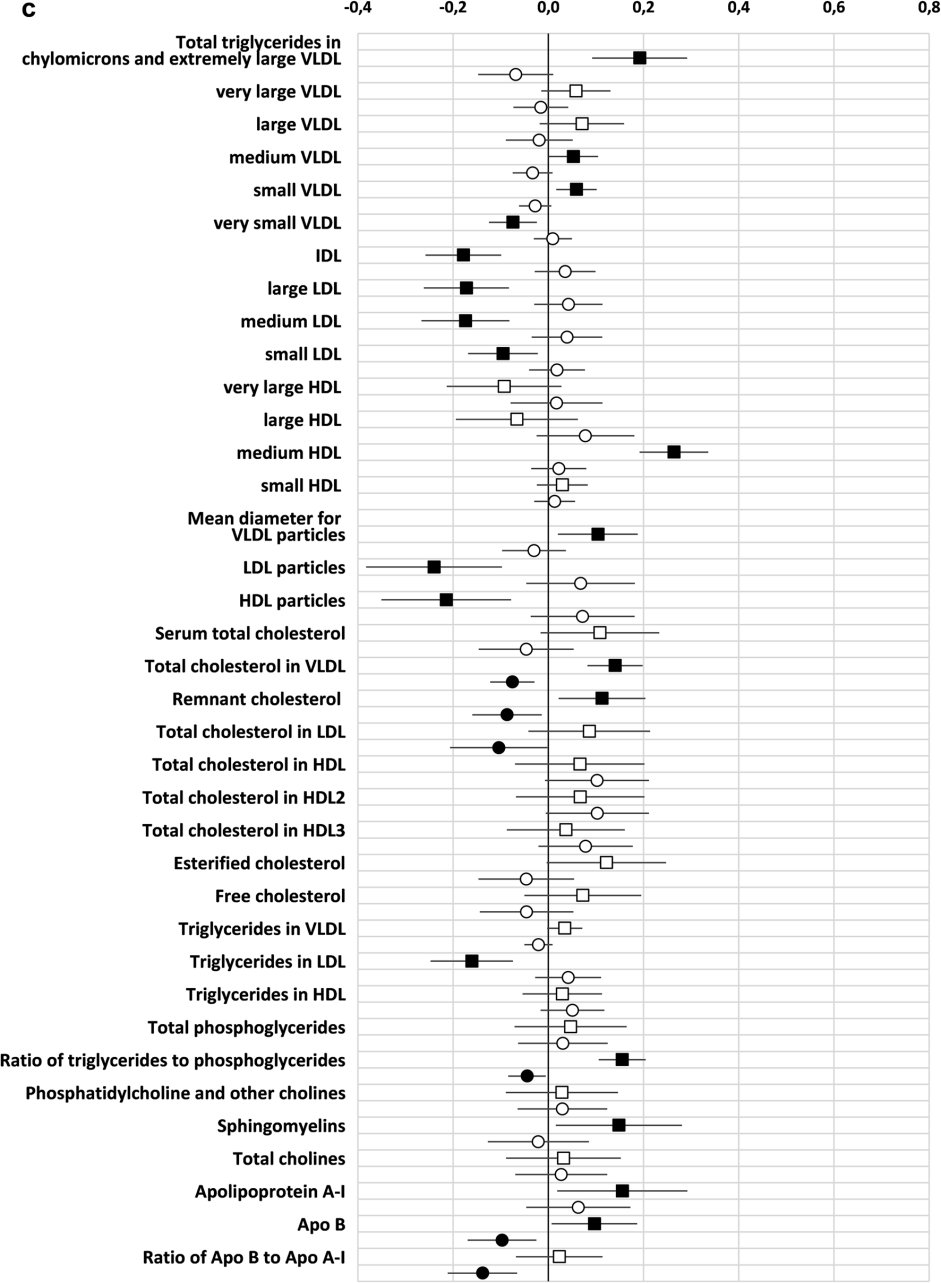

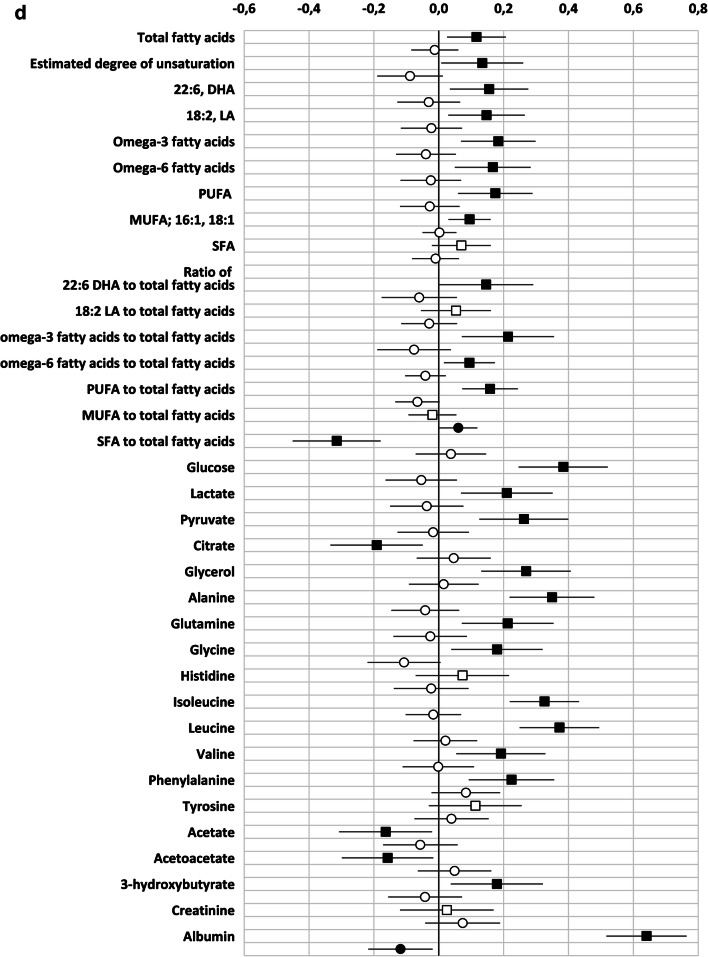
**a-d** Unstandardized beta (95% CI) of the linear regression between GlycA (squares) and hsCRP (circles) with metabolic variables. Both inflammatory markers and the metabolites are divided by their standard deviation. Black squares/circles indicate statistically significant correlation between inflammatory marker and metabolic variables (P < 0.05). *DHA* docosahexaenoic acid, *FA* fatty acids, *LA* linoleic acids, *MUFA* monounsaturated fatty acids, *PUFA* polyunsaturated fatty acids; Remnant cholesterol (non-HDL, non-LDL -cholesterol)

As observed in the correlation analysis, GlycA was better than hsCRP in reflecting the metabolomics markers. We observed 90/156 significant associations with GlycA but only 32 with hsCRP (Supplementary Table 1, Fig. 2). Regarding both inflammatory markers, the direct correlation between the concentration and the constituents of medium HDL particles remained statistically significant. Several correlations were only observed between lipid variables and GlycA, including direct correlations with the concentration and the constituents of several sized VLDL- and small HDL-particles and an inverse correlation between the concentration and the constituents of very large HDL-particles. In this multilinear regression model, some of the associations between hsCRP and VLDL-particles and their constituents were negative. This is at odds with the findings observed in the Pearson correlation and may be due to the interference of the strong correlations between GlycA and triglycerides and the VLDL- particles and their constituents. With respect to the amino acids, the correlation between the concentration of GlycA, but not that of hsCRP, and the amino acids isoleucine, glycine, leucine, valine and phenylalanine (Supplementary Table 1, Fig. 2), remained significant in the multiple linear model. When focusing on gut microbiome diversity, the indirect correlation between GlycA and diversity was also statistically significant, while no correlation was seen with hsCRP (Table 2). When the intake of dietary fibre was taken into account, the correlation between MGS richness and GlycA remained statistically significant (B (95% CI): − 0.195 (− 20.6; − 2.7), P = 0.011, adjusted R^2^ = 0.042), and a trend in correlation between Gene richness (B (95% CI − 16,523.5 (− 33,060.1; 13.0), P = 0.050, adjusted R^2^ = 0.040) and MGS Shannon (B (95% CI − 0.047 (− 0.094; 0.001), P = 0.053, adjusted R^2^ = 0.027) and GlycA were detected.

**Table 2:** Multiple linear regression describing the relationship between gut microbiome diversity and GlycA and hsCRP

	B (95% CI)	P value	Adjusted R^2^
Gene Shannon			0.032
GlycA	− 0.129 (− 0.236; − 0.021)	0.019	
hsCRP	0.017 (− 0.069; 0.103)	0.635	
Gene richness			0.048
GlycA	− 20,364.0 (− 36,123.1; − 4604.9)	0.011	
hsCRP	1966.1 (− 10,642.8; 14,574.9)	0.759	
MGS Shannon			0.036
GlycA	− 0.056 (− 0.101; − 0.011)	0.015	
hsCRP	0.009 (− 0.028; 0.045)	0.639	
MGS richness			0.046
GlycA	− 13.2 (− 21.8; − 4.7)	0.003	
hsCRP	1.1 (− 5.8; 7.9)	0.755	

The adjusted R-squared value is for the whole model including also prepregnancy BMI, serum triglycerides and gestational weeks

No correlations were observed between concentrations of GlycA or hsCRP with the level of LPS (P < 0.190 for both) in the multiple linear regression when adjusted with values of prepregnancy BMI, serum triglycerides and gestational weeks.

## Discussion

We demonstrated the superiority of using the low grade inflammatory marker, GlycA, over hsCRP in reflecting the host’s metabolic status in overweight and obese pregnant women. The associations observed between GlycA and metabolomic markers were specifically related to those lipoproteins linked previously with the risk of cardiovascular diseases (Kontush 2015; Sacks et al. 2000) and amino acids, such as the BCAAs, which have been associated with type 2 diabetes (Yoon 2016). In addition, higher levels of GlycA, but not hsCRP, associated with a lower gut microbiome diversity, which indicates that GlycA may also be considered as a marker for gut microbiota dysbiosis, a phenomenon observed in clinical conditions manifested with metabolic aberrations*.* In summary, our findings suggest that GlycA may be considered as a more sensitive marker in reflecting and identifying subjects at risk for developing metabolic complications.

This is the first study that has compared GlycA with hsCRP in relation to serum metabolomics in overweight and obese pregnant women; it was found that the former correlates with more metabolomics variables and thus may be a more sensitive marker of an disturbed metabolomic profile in those individuals at risk for clinical complications, particularly cardiovascular diseases. Previous studies have reported a clearer correlation between GlycA and clinically relevant measures such as triglycerides and HDL- and LDL- cholesterol (Fizelova et al. 2017) in comparison to the traditional biochemical marker, hsCRP. Furthermore, when compared to hsCRP, GlycA has shown to be a better predictor for cardiometabolic diseases (Connelley et al. 2017). These findings support our proposal that GlycA is a more reliable marker for metabolic complications than hsCRP.

Adipose tissue is known to be a major contributor to low grade inflammation (Calder et al. 2011). Both inflammatory markers associated with BMI in our participants, as has been the case in previous studies (e.g. Otvos et al. 2015; Lorenzo et al. 2017; Fizelova et al. 2017). Nevertheless, in order that our investigation of the relationship between metabolomics and inflammatory markers would be independent of obesity, we included prepregnancy BMI in the analyses. Subsequently, several relationships were detected, suggesting that obesity is only one explanatory factor and thus only partially explained the observed relations. We detected a weak, but significant inverse correlation between GlycA and gut microbiome diversity, while no relationship was evident between hsCRP and gut microbiome diversity*.* In addition, the GlycA level was related to higher serum LPS, further pointing to the involvement of gut microbiota in low grade inflammation. However, this correlation no longer remained statistically significant when pprepregnancy BMI, serum triglycerides and gestational weeks were taken into account in the analysis.

In this study we have focused on overweight and obese pregnant women, which is an important study population due to their increased risk for metabolic diseases. The strength of the study is, that we have utilized high-tech approaches for analyzing both the serum metabolomics and gut microbiome diversity, which compared to mostly used 16S rRNA method, provides more information on gut microbiome diversity, i.e. the diversity and richness of the genes.In addition, we have applied robust statistical methods, i.e. adjusted the analysis with values of prepregnancy BMI, serum triglycerides and gestational weeks to evaluate the independent effects of GlycA and hsCRP. It is of note that the faecal sample collection method may induce a limitation as the variation in individual collection times may induce a batch effect on our metagenomics data. Further, our study is limited to a population of overweight and obese pregnant women. However, our GlycA values measured in early pregnancy were similar or even lower compared to values in previous study in a Finnish population (Ritchie et al. 2015). We thus anticipate that the findings presented here could be comparable to normal weight and non-pregnant women, but this will need to be confirmed as well as it's clinical relevance evaluated.

Conclusions. GlycA, a novel marker of low grade inflammation, reflected gut microbiome diversity and also the complex host metabolic status in a more robust way than hsCRP, a traditional marker for low grade inflammation. Thus, GlycA may be a more feasible marker for identifying the metabolic aberrations in overweight and obese women, i.e. a group of individuals at risk of developing clinical metabolic disorders.

## Electronic supplementary material

Below is the link to the electronic supplementary material.

Supplementary file1 (DOCX 29 kb)

Supplementary file2 Supplemental table 1. Unstandardized B-values of GlycA and hsCRP from multiple linear regression with GlycA, hsCRP, prepregnancy BMI, serum triglycerides and gestational weeks. Adjusted R squared value is for the whole model. (PDF 732 kb)

## Data Availability

The data are not publicly available due to them containing information that could compromise research participant privacy/consent.

## Abbreviations

BH-procedure: Benjamini–Hochberg-procedure
BMI: Body mass index
FDR: False discovery rate
GlycA: Glycoprotein acetylation
hsCRP: High sensitivity C-reactive protein
DL: High density lipoprotein
IGC: Integrated gene catalog
LPS: Lipopolysaccharide
MGS: Metagenomic species
qPCR: Quantitative real-time PCR
TG: Triglycerides
VLDL: Very low density lipoprotein
