# Supplementary material for: GlycA, a novel marker for low grade inflammation, reflects gut microbiome diversity and is more accurate than high sensitive CRP in reflecting metabolomic profile
Source: Metabolomics. 2020 Jun 20;16(7):76. doi: 10.1007/s11306-020-01695-x (PMC7306047; doi:10.1007/s11306-020-01695-x)
Supplement: Supplementary file 2 — Supplementary file2 Supplemental table 1. Unstandardized B-values of GlycA and hsCRP from multiple linear regression with GlycA, hsCRP, prepregnancy BMI, serum triglycerides and gestational weeks. Adjusted R squared value is for the whole model. (PDF 732 kb) [file 11306_2020_1695_MOESM2_ESM.pdf]

		Unstandardized	95,0% Confidence Interval for B			
		B	Lower Bound	Upper Bound	P-value	Adjusted R Square
Concentration of chylomicrons and extremely large VLDL particles	GlycA	0,172	0,081	0,263	0,000	0,637
	Triglycerides	0,781	0,687	0,876	0,000	
	hsCRP	-0,061	-0,133	0,011	0,095	
	prepregnancy	0,008	-0,065	0,080	0,834	
	Gestational	-0,191	-0,262	-0,119	0,000	
Concentration of very large VLDL particles	GlycA	0,071	0,004	0,139	0,039	0,811
	Triglycerides	1,020	0,946	1,093	0,000	
	hsCRP	-0,025	-0,078	0,029	0,365	
	prepregnancy	0,017	-0,037	0,071	0,544	
	Gestational	-0,189	-0,242	-0,136	0,000	
Concentration of large VLDL particles	GlycA	0,088	0,005	0,172	0,039	0,811
	Triglycerides	1,262	1,172	1,353	0,000	
	hsCRP	-0,031	-0,097	0,036	0,365	
	prepregnancy	0,021	-0,046	0,088	0,544	
	Gestational	-0,234	-0,299	-0,168	0,000	
Concentration of medium VLDL particles	GlycA	0,073	0,035	0,112	0,000	0,928
	Triglycerides	0,950	0,912	0,987	0,000	
	hsCRP	-0,038	-0,069	-0,007	0,016	
	prepregnancy	0,010	-0,021	0,041	0,512	
	Gestational	-0,113	-0,144	-0,082	0,000	
Concentration of small VLDL particles	GlycA	0,104	0,064	0,144	0,000	0,925
	Triglycerides	0,896	0,857	0,934	0,000	
	hsCRP	-0,039	-0,071	-0,007	0,016	
	prepregnancy	0,010	-0,022	0,042	0,540	
	Gestational	0,013	-0,018	0,045	0,414	
Concentration of very small VLDL particles	GlycA	0,004	-0,088	0,095	0,937	0,602
	Triglycerides	0,721	0,632	0,810	0,000	
	hsCRP	-0,047	-0,120	0,027	0,211	
	prepregnancy	0,006	-0,067	0,079	0,871	
	Gestational	0,160	0,087	0,232	0,000	
Concentration of IDL particles	GlycA	0,013	-0,104	0,130	0,825	0,347
	Triglycerides	0,511	0,397	0,625	0,000	
	hsCRP	-0,068	-0,162	0,026	0,155	
	prepregnancy	-0,028	-0,122	0,066	0,556	
	Gestational	0,198	0,105	0,291	0,000	
Concentration of large LDL particles	GlycA	0,053	-0,068	0,173	0,390	0,309
	Triglycerides	0,454	0,336	0,571	0,000	
	hsCRP	-0,082	-0,178	0,014	0,095	
	prepregnancy	-0,035	-0,131	0,062	0,481	
	Gestational	0,196	0,101	0,292	0,000	
Concentration of medium LDL particles	GlycA	0,070	-0,054	0,194	0,268	0,293
	Triglycerides	0,442	0,322	0,563	0,000	
	hsCRP	-0,095	-0,194	0,005	0,062	
	prepregnancy	-0,036	-0,135	0,064	0,479	
	Gestational	0,189	0,090	0,287	0,000	
Concentration of small LDL particles	GlycA	0,070	-0,052	0,192	0,261	0,288
	Triglycerides	0,425	0,306	0,544	0,000	
	hsCRP	-0,085	-0,183	0,013	0,089	
	prepregnancy	-0,046	-0,144	0,052	0,358	
	Gestational	0,192	0,095	0,289	0,000	
Concentration of very large HDL particles	GlycA	-0,195	-0,333	-0,058	0,005	0,100
	Triglycerides	0,029	-0,105	0,163	0,667	
	hsCRP	0,068	-0,042	0,178	0,225	
	prepregnancy	-0,151	-0,261	-0,040	0,008	
	Gestational	0,244	0,135	0,353	0,000	
Concentration of large HDL particles	GlycA	-0,055	-0,192	0,082	0,427	0,104
	Triglycerides	-0,144	-0,278	-0,011	0,034	
	hsCRP	0,112	0,002	0,222	0,045	
	prepregnancy	-0,159	-0,269	-0,049	0,005	

	Gestational	0,261	0,153	0,370	0,000	
Concentration of medium HDL particles	GlycA	0,391	0,257	0,526	0,000	0,131
	Triglycerides	-0,257	-0,388	-0,125	0,000	
	hsCRP	0,113	0,005	0,221	0,040	
	prepregnancy	-0,036	-0,144	0,072	0,512	
	Gestational	0,140	0,033	0,247	0,010	
Concentration of small HDL particles	GlycA	0,473	0,346	0,600	0,000	0,232
	Triglycerides	-0,083	-0,206	0,041	0,189	
	hsCRP	0,057	-0,045	0,158	0,273	
	prepregnancy	0,073	-0,029	0,175	0,160	
	Gestational	0,078	-0,022	0,179	0,126	
Total lipids in chylomicrons and extremely large VLDL particles	GlycA	0,164	0,075	0,252	0,000	0,659
	Triglycerides	0,802	0,711	0,894	0,000	
	hsCRP	-0,058	-0,127	0,012	0,104	
	prepregnancy	0,004	-0,066	0,074	0,917	
	Gestational	-0,182	-0,251	-0,113	0,000	
Total lipids in very large VLDL particles	GlycA	0,075	0,008	0,142	0,028	0,815
	Triglycerides	1,021	0,948	1,093	0,000	
	hsCRP	-0,027	-0,080	0,026	0,312	
	prepregnancy	0,013	-0,040	0,067	0,630	
	Gestational	-0,184	-0,237	-0,132	0,000	
Total lipids in large VLDL particles	GlycA	0,093	0,010	0,175	0,028	0,815
	Triglycerides	1,262	1,173	1,352	0,000	
	hsCRP	-0,034	-0,099	0,032	0,312	
	prepregnancy	0,016	-0,050	0,082	0,630	
	Gestational	-0,228	-0,293	-0,163	0,000	
Total lipids in medium VLDL particles	GlycA	0,075	0,040	0,111	0,000	0,939
	Triglycerides	0,953	0,918	0,988	0,000	
	hsCRP	-0,038	-0,066	-0,009	0,010	
	prepregnancy	0,006	-0,022	0,035	0,667	
	Gestational	-0,105	-0,134	-0,077	0,000	
Total lipids in small VLDL particles	GlycA	0,111	0,069	0,152	0,000	0,918
	Triglycerides	0,884	0,844	0,925	0,000	
	hsCRP	-0,041	-0,074	-0,007	0,017	
	prepregnancy	0,008	-0,025	0,042	0,622	
	Gestational	0,025	-0,008	0,058	0,136	
Total lipids in very small VLDL particles	GlycA	0,013	-0,083	0,110	0,784	0,556
	Triglycerides	0,683	0,589	0,777	0,000	
	hsCRP	-0,051	-0,129	0,026	0,192	
	prepregnancy	0,003	-0,075	0,080	0,942	
	Gestational	0,166	0,089	0,242	0,000	
Total lipids in IDL particles	GlycA	0,023	-0,096	0,143	0,701	0,314
	Triglycerides	0,476	0,359	0,593	0,000	
	hsCRP	-0,072	-0,168	0,024	0,140	
	prepregnancy	-0,031	-0,127	0,065	0,525	
	Gestational	0,197	0,102	0,292	0,000	
Total lipids in large LDL particles	GlycA	0,059	-0,063	0,181	0,343	0,286
	Triglycerides	0,429	0,310	0,548	0,000	
	hsCRP	-0,088	-0,186	0,010	0,078	
	prepregnancy	-0,035	-0,133	0,063	0,484	
	Gestational	0,194	0,097	0,291	0,000	
Total lipids in medium LDL particles	GlycA	0,075	-0,048	0,199	0,228	0,277
	Triglycerides	0,416	0,297	0,536	0,000	
	hsCRP	-0,096	-0,195	0,002	0,055	
	prepregnancy	-0,036	-0,135	0,062	0,468	
	Gestational	0,183	0,085	0,281	0,000	
Total lipids in small LDL particles	GlycA	0,075	-0,049	0,199	0,235	0,269
	Triglycerides	0,403	0,283	0,524	0,000	
	hsCRP	-0,087	-0,186	0,012	0,085	
	prepregnancy	-0,047	-0,146	0,052	0,354	
	Gestational	0,191	0,093	0,290	0,000	
Total lipids in very large HDL particles	GlycA	-0,195	-0,332	-0,058	0,005	0,100
	Triglycerides	0,025	-0,109	0,159	0,716	

	hsCRP	0,068	-0,042	0,178	0,224	
	prepregnancy	-0,150	-0,260	-0,040	0,008	
	Gestational	0,244	0,135	0,353	0,000	
Total lipids in large HDL particles	GlycA	-0,063	-0,199	0,074	0,369	0,108
	Triglycerides	-0,155	-0,288	-0,021	0,023	
	hsCRP	0,113	0,003	0,222	0,044	
	prepregnancy	-0,160	-0,270	-0,050	0,004	
	Gestational	0,260	0,151	0,368	0,000	
Total lipids in medium HDL particles	GlycA	0,392	0,257	0,527	0,000	0,130
	Triglycerides	-0,287	-0,419	-0,156	0,000	
	hsCRP	0,113	0,005	0,221	0,041	
	prepregnancy	-0,040	-0,148	0,068	0,469	
	Gestational	0,143	0,036	0,250	0,009	
Total lipids in small HDL particles	GlycA	0,480	0,352	0,608	0,000	0,221
	Triglycerides	-0,116	-0,240	0,008	0,068	
	hsCRP	0,055	-0,048	0,157	0,295	
	prepregnancy	0,068	-0,035	0,170	0,194	
	Gestational	0,087	-0,015	0,188	0,093	
Phospholipids in chylomicrons and extremely large VLDL particles	GlycA	0,157	0,070	0,243	0,000	0,674
	Triglycerides	0,811	0,722	0,901	0,000	
	hsCRP	-0,019	-0,087	0,049	0,585	
	prepregnancy	-0,033	-0,102	0,035	0,340	
	Gestational	-0,127	-0,195	-0,060	0,000	
Phospholipids in very large VLDL particles	GlycA	0,167	0,092	0,243	0,000	0,763
	Triglycerides	0,927	0,844	1,010	0,000	
	hsCRP	-0,054	-0,114	0,006	0,076	
	prepregnancy	0,005	-0,056	0,066	0,865	
	Gestational	-0,145	-0,205	-0,086	0,000	
Phospholipids in large VLDL particles	GlycA	0,252	0,138	0,366	0,000	0,763
	Triglycerides	1,397	1,272	1,522	0,000	
	hsCRP	-0,082	-0,172	0,009	0,076	
	prepregnancy	0,008	-0,084	0,100	0,865	
	Gestational	-0,219	-0,309	-0,129	0,000	
Phospholipids in medium VLDL particles	GlycA	0,086	0,052	0,119	0,000	0,946
	Triglycerides	0,946	0,913	0,978	0,000	
	hsCRP	-0,038	-0,064	-0,011	0,006	
	prepregnancy	0,007	-0,020	0,034	0,616	
	Gestational	-0,087	-0,113	-0,060	0,000	
Phospholipids in small VLDL particles	GlycA	0,097	0,053	0,140	0,000	0,909
	Triglycerides	0,872	0,830	0,915	0,000	
	hsCRP	-0,012	-0,047	0,023	0,514	
	prepregnancy	0,021	-0,014	0,056	0,239	
	Gestational	0,052	0,018	0,087	0,003	
Phospholipids in very small VLDL particles	GlycA	0,005	-0,108	0,117	0,936	0,395
	Triglycerides	0,561	0,451	0,671	0,000	
	hsCRP	-0,060	-0,150	0,031	0,195	
	prepregnancy	-0,005	-0,096	0,085	0,907	
	Gestational	0,186	0,097	0,276	0,000	
Phospholipids in IDL particles	GlycA	0,027	-0,098	0,152	0,674	0,254
	Triglycerides	0,418	0,296	0,540	0,000	
	hsCRP	-0,080	-0,180	0,020	0,116	
	prepregnancy	-0,036	-0,136	0,064	0,482	
	Gestational	0,193	0,093	0,292	0,000	
Phospholipids in large LDL particles	GlycA	0,087	-0,036	0,210	0,167	0,276
	Triglycerides	0,408	0,288	0,528	0,000	
	hsCRP	-0,089	-0,187	0,010	0,077	
	prepregnancy	-0,034	-0,133	0,065	0,500	
	Gestational	0,183	0,085	0,280	0,000	
Phospholipids in medium LDL particles	GlycA	0,101	-0,014	0,217	0,084	0,368
	Triglycerides	0,493	0,381	0,606	0,000	
	hsCRP	-0,087	-0,179	0,005	0,064	
	prepregnancy	-0,024	-0,116	0,068	0,608	
	Gestational	0,161	0,070	0,253	0,001	

Phospholipids in small LDL particles	GlycA	0,105	-0,013	0,223	0,080	0,336
	Triglycerides	0,445	0,330	0,560	0,000	
	hsCRP	-0,060	-0,154	0,034	0,213	
	prepregnancy	-0,044	-0,139	0,051	0,362	
	Gestational	0,190	0,096	0,284	0,000	
Phospholipids in very large HDL particles	GlycA	-0,203	-0,339	-0,066	0,004	0,110
	Triglycerides	-0,028	-0,161	0,105	0,675	
	hsCRP	0,080	-0,030	0,189	0,153	
	prepregnancy	-0,148	-0,258	-0,038	0,008	
	Gestational	0,247	0,138	0,355	0,000	
Phospholipids in large HDL particles	GlycA	-0,014	-0,151	0,123	0,840	0,107
	Triglycerides	-0,184	-0,318	-0,051	0,007	
	hsCRP	0,116	0,006	0,225	0,038	
	prepregnancy	-0,153	-0,262	-0,043	0,007	
	Gestational	0,268	0,159	0,376	0,000	
Phospholipids in medium HDL particles	GlycA	0,327	0,192	0,462	0,000	0,131
	Triglycerides	-0,161	-0,293	-0,030	0,016	
	hsCRP	0,129	0,021	0,237	0,020	
	prepregnancy	-0,042	-0,150	0,066	0,444	
	Gestational	0,183	0,076	0,290	0,001	
Phospholipids in small HDL particles	GlycA	0,418	0,285	0,551	0,000	0,157
	Triglycerides	-0,134	-0,264	-0,005	0,042	
	hsCRP	0,094	-0,013	0,200	0,084	
	prepregnancy	0,065	-0,042	0,171	0,235	
	Gestational	-0,007	-0,113	0,098	0,895	
Total cholesterol in chylomicrons and extremely large VLDL particles	GlycA	0,075	-0,004	0,154	0,062	0,731
	Triglycerides	0,905	0,823	0,986	0,000	
	hsCRP	-0,025	-0,087	0,036	0,419	
	prepregnancy	-0,051	-0,113	0,012	0,111	
	Gestational	-0,094	-0,156	-0,033	0,003	
Total cholesterol in very large VLDL particles	GlycA	0,037	-0,038	0,112	0,329	0,768
	Triglycerides	1,023	0,941	1,104	0,000	
	hsCRP	-0,041	-0,100	0,019	0,180	
	prepregnancy	-0,020	-0,080	0,040	0,522	
	Gestational	-0,160	-0,219	-0,101	0,000	
Total cholesterol in large VLDL particles	GlycA	0,049	-0,049	0,146	0,329	0,768
	Triglycerides	1,333	1,227	1,438	0,000	
	hsCRP	-0,053	-0,130	0,024	0,180	
	prepregnancy	-0,025	-0,104	0,053	0,522	
	Gestational	-0,208	-0,285	-0,132	0,000	
Total cholesterol in medium VLDL particles	GlycA	0,097	0,062	0,133	0,000	0,940
	Triglycerides	0,931	0,896	0,965	0,000	
	hsCRP	-0,041	-0,070	-0,013	0,004	
	prepregnancy	-0,024	-0,052	0,005	0,101	
	Gestational	-0,044	-0,072	-0,016	0,002	
Total cholesterol in small VLDL particles	GlycA	0,171	0,101	0,241	0,000	0,765
	Triglycerides	0,745	0,677	0,814	0,000	
	hsCRP	-0,068	-0,124	-0,012	0,018	
	prepregnancy	-0,009	-0,065	0,047	0,752	
	Gestational	0,083	0,028	0,139	0,003	
Total cholesterol in very small VLDL particles	GlycA	0,066	-0,047	0,178	0,253	0,392
	Triglycerides	0,531	0,421	0,641	0,000	
	hsCRP	-0,072	-0,162	0,019	0,119	
	prepregnancy	0,001	-0,090	0,092	0,983	
	Gestational	0,166	0,077	0,256	0,000	
Total cholesterol in IDL particles	GlycA	0,061	-0,064	0,187	0,335	0,251
	Triglycerides	0,399	0,277	0,521	0,000	
	hsCRP	-0,088	-0,188	0,013	0,087	
	prepregnancy	-0,035	-0,135	0,066	0,494	
	Gestational	0,184	0,084	0,283	0,000	
Total cholesterol in large LDL particles	GlycA	0,081	-0,045	0,208	0,208	0,237
	Triglycerides	0,373	0,250	0,496	0,000	
	hsCRP	-0,102	-0,204	-0,001	0,048	

	prepregnancy	-0,037	-0,139	0,064	0,470	
	Gestational	0,180	0,079	0,280	0,000	
Total cholesterol in medium LDL particles	GlycA	0,094	-0,034	0,223	0,149	0,212
	Triglycerides	0,340	0,215	0,466	0,000	
	hsCRP	-0,111	-0,214	-0,008	0,034	
	prepregnancy	-0,041	-0,144	0,063	0,439	
	Gestational	0,174	0,072	0,276	0,001	
Total cholesterol in small LDL particles	GlycA	0,081	-0,049	0,212	0,219	0,192
	Triglycerides	0,318	0,191	0,445	0,000	
	hsCRP	-0,104	-0,208	0,000	0,050	
	prepregnancy	-0,047	-0,152	0,057	0,375	
	Gestational	0,183	0,079	0,286	0,001	
Total cholesterol in very large HDL particles	GlycA	-0,190	-0,327	-0,052	0,007	0,095
	Triglycerides	0,043	-0,091	0,177	0,529	
	hsCRP	0,057	-0,053	0,167	0,311	
	prepregnancy	-0,150	-0,260	-0,039	0,008	
	Gestational	0,238	0,129	0,348	0,000	
Total cholesterol in large HDL particles	GlycA	-0,096	-0,232	0,039	0,162	0,123
	Triglycerides	-0,183	-0,315	-0,051	0,007	
	hsCRP	0,110	0,001	0,219	0,047	
	prepregnancy	-0,163	-0,272	-0,055	0,003	
	Gestational	0,249	0,141	0,356	0,000	
Total cholesterol in medium HDL particles	GlycA	0,414	0,281	0,547	0,000	0,161
	Triglycerides	-0,484	-0,614	-0,355	0,000	
	hsCRP	0,098	-0,008	0,205	0,069	
	prepregnancy	-0,044	-0,151	0,062	0,413	
	Gestational	0,111	0,006	0,216	0,039	
Total cholesterol in small HDL particles	GlycA	0,449	0,317	0,582	0,000	0,158
	Triglycerides	-0,269	-0,398	-0,139	0,000	
	hsCRP	-0,010	-0,117	0,096	0,846	
	prepregnancy	0,039	-0,068	0,145	0,474	
	Gestational	0,191	0,085	0,296	0,000	
Cholesterol esters in chylomicrons and extremely large VLDL particles	GlycA	0,032	-0,065	0,130	0,515	0,586
	Triglycerides	0,838	0,736	0,939	0,000	
	hsCRP	-0,043	-0,120	0,034	0,271	
	prepregnancy	-0,021	-0,101	0,058	0,595	
	Gestational	-0,092	-0,168	-0,015	0,019	
Cholesterol esters in very large VLDL particles	GlycA	-0,037	-0,118	0,043	0,362	0,732
	Triglycerides	1,049	0,962	1,136	0,000	
	hsCRP	-0,044	-0,108	0,020	0,174	
	prepregnancy	-0,018	-0,083	0,046	0,575	
	Gestational	-0,174	-0,237	-0,111	0,000	
Cholesterol esters in large VLDL particles	GlycA	-0,052	-0,166	0,061	0,362	0,732
	Triglycerides	1,477	1,354	1,600	0,000	
	hsCRP	-0,062	-0,152	0,028	0,174	
	prepregnancy	-0,026	-0,117	0,065	0,575	
	Gestational	-0,245	-0,334	-0,156	0,000	
Cholesterol esters in medium VLDL particles	GlycA	0,163	0,109	0,216	0,000	0,863
	Triglycerides	0,840	0,787	0,892	0,000	
	hsCRP	-0,085	-0,128	-0,043	0,000	
	prepregnancy	-0,021	-0,064	0,022	0,345	
	Gestational	-0,006	-0,049	0,037	0,784	
Cholesterol esters in small VLDL particles	GlycA	0,213	0,125	0,301	0,000	0,631
	Triglycerides	0,640	0,554	0,725	0,000	
	hsCRP	-0,098	-0,169	-0,028	0,006	
	prepregnancy	-0,013	-0,084	0,057	0,714	
	Gestational	0,078	0,008	0,148	0,028	
Cholesterol esters in very small VLDL particles	GlycA	0,083	-0,032	0,198	0,156	0,369
	Triglycerides	0,510	0,398	0,622	0,000	
	hsCRP	-0,085	-0,177	0,007	0,071	
	prepregnancy	0,021	-0,071	0,113	0,658	
	Gestational	0,146	0,055	0,237	0,002	
Cholesterol esters in	GlycA	0,080	-0,042	0,203	0,197	0,287

IDL particles	Triglycerides	0,431	0,312	0,550	0,000	
	hsCRP	-0,093	-0,191	0,005	0,063	
	prepregnancy	-0,027	-0,125	0,071	0,593	
	Gestational	0,168	0,071	0,265	0,001	
Cholesterol esters in large LDL particles	GlycA	0,093	-0,031	0,218	0,142	0,258
	Triglycerides	0,393	0,272	0,515	0,000	
	hsCRP	-0,107	-0,206	-0,007	0,037	
	prepregnancy	-0,034	-0,134	0,066	0,502	
	Gestational	0,171	0,072	0,270	0,001	
Cholesterol esters in medium LDL particles	GlycA	0,099	-0,030	0,228	0,134	0,204
	Triglycerides	0,331	0,205	0,457	0,000	
	hsCRP	-0,116	-0,219	-0,013	0,028	
	prepregnancy	-0,039	-0,143	0,064	0,458	
	Gestational	0,168	0,065	0,270	0,001	
Cholesterol esters in small LDL particles	GlycA	0,081	-0,051	0,212	0,228	0,175
	Triglycerides	0,304	0,176	0,432	0,000	
	hsCRP	-0,113	-0,218	-0,007	0,036	
	prepregnancy	-0,042	-0,147	0,064	0,437	
	Gestational	0,174	0,069	0,278	0,001	
Cholesterol esters in very large HDL particles	GlycA	-0,190	-0,328	-0,053	0,007	0,095
	Triglycerides	0,053	-0,081	0,187	0,439	
	hsCRP	0,053	-0,057	0,164	0,342	
	prepregnancy	-0,150	-0,261	-0,040	0,008	
	Gestational	0,236	0,127	0,346	0,000	
Cholesterol esters in large HDL particles	GlycA	-0,093	-0,229	0,043	0,180	0,118
	Triglycerides	-0,176	-0,308	-0,044	0,009	
	hsCRP	0,112	0,003	0,221	0,044	
	prepregnancy	-0,162	-0,271	-0,053	0,004	
	Gestational	0,249	0,141	0,357	0,000	
Cholesterol esters in medium HDL particles	GlycA	0,426	0,295	0,558	0,000	0,178
	Triglycerides	-0,529	-0,657	-0,401	0,000	
	hsCRP	0,091	-0,014	0,196	0,090	
	prepregnancy	-0,040	-0,145	0,066	0,458	
	Gestational	0,090	-0,014	0,194	0,090	
Cholesterol esters in small HDL particles	GlycA	0,395	0,260	0,531	0,000	0,122
	Triglycerides	-0,269	-0,401	-0,137	0,000	
	hsCRP	-0,042	-0,151	0,066	0,444	
	prepregnancy	0,045	-0,064	0,154	0,418	
	Gestational	0,193	0,085	0,300	0,000	
Free cholesterol in chylomicrons and extremely large VLDL particles	GlycA	0,089	0,014	0,165	0,021	0,751
	Triglycerides	0,898	0,820	0,977	0,000	
	hsCRP	0,003	-0,056	0,063	0,916	
	prepregnancy	-0,023	-0,083	0,037	0,448	
	Gestational	-0,101	-0,160	-0,042	0,001	
Free cholesterol in very large VLDL particles	GlycA	0,121	0,038	0,203	0,004	0,719
	Triglycerides	0,934	0,844	1,024	0,000	
	hsCRP	-0,028	-0,094	0,037	0,396	
	prepregnancy	-0,027	-0,093	0,039	0,422	
	Gestational	-0,141	-0,206	-0,076	0,000	
Free cholesterol in large VLDL particles	GlycA	0,147	0,046	0,248	0,004	0,719
	Triglycerides	1,139	1,030	1,249	0,000	
	hsCRP	-0,035	-0,114	0,045	0,396	
	prepregnancy	-0,033	-0,114	0,048	0,422	
	Gestational	-0,172	-0,251	-0,093	0,000	
Free cholesterol in medium VLDL particles	GlycA	0,048	0,022	0,075	0,000	0,967
	Triglycerides	0,974	0,949	1,000	0,000	
	hsCRP	-0,014	-0,036	0,007	0,181	
	prepregnancy	-0,014	-0,035	0,007	0,203	
	Gestational	-0,060	-0,081	-0,039	0,000	
Free cholesterol in small VLDL particles	GlycA	0,091	0,047	0,136	0,000	0,906
	Triglycerides	0,866	0,823	0,909	0,000	
	hsCRP	-0,015	-0,050	0,021	0,413	
	prepregnancy	-0,003	-0,039	0,033	0,870	

	Gestational	0,087	0,052	0,122	0,000	
Free cholesterol in very small VLDL particles	GlycA	0,032	-0,079	0,143	0,569	0,415
	Triglycerides	0,553	0,445	0,660	0,000	
	hsCRP	-0,044	-0,133	0,045	0,329	
	prepregnancy	-0,037	-0,126	0,052	0,413	
	Gestational	0,201	0,113	0,289	0,000	
Free cholesterol in IDL particles	GlycA	0,013	-0,119	0,145	0,846	0,174
	Triglycerides	0,313	0,185	0,441	0,000	
	hsCRP	-0,073	-0,179	0,032	0,173	
	prepregnancy	-0,056	-0,161	0,050	0,300	
	Gestational	0,217	0,112	0,321	0,000	
Free cholesterol in large LDL particles	GlycA	0,045	-0,087	0,176	0,505	0,178
	Triglycerides	0,308	0,181	0,436	0,000	
	hsCRP	-0,088	-0,193	0,017	0,102	
	prepregnancy	-0,046	-0,151	0,059	0,392	
	Gestational	0,205	0,101	0,310	0,000	
Free cholesterol in medium LDL particles	GlycA	0,075	-0,052	0,201	0,246	0,237
	Triglycerides	0,368	0,245	0,491	0,000	
	hsCRP	-0,090	-0,191	0,011	0,081	
	prepregnancy	-0,045	-0,147	0,056	0,379	
	Gestational	0,193	0,093	0,294	0,000	
Free cholesterol in small LDL particles	GlycA	0,081	-0,045	0,207	0,204	0,244
	Triglycerides	0,359	0,236	0,481	0,000	
	hsCRP	-0,069	-0,170	0,032	0,180	
	prepregnancy	-0,066	-0,167	0,035	0,199	
	Gestational	0,208	0,109	0,308	0,000	
Free cholesterol in very large HDL particles	GlycA	-0,187	-0,325	-0,050	0,008	0,096
	Triglycerides	0,019	-0,115	0,153	0,780	
	hsCRP	0,065	-0,045	0,175	0,248	
	prepregnancy	-0,148	-0,258	-0,037	0,009	
	Gestational	0,241	0,132	0,350	0,000	
Free cholesterol in large HDL particles	GlycA	-0,108	-0,242	0,027	0,116	0,137
	Triglycerides	-0,207	-0,338	-0,076	0,002	
	hsCRP	0,104	-0,003	0,212	0,057	
	prepregnancy	-0,167	-0,275	-0,059	0,002	
	Gestational	0,248	0,142	0,355	0,000	
Free cholesterol in medium HDL particles	GlycA	0,350	0,215	0,485	0,000	0,127
	Triglycerides	-0,302	-0,433	-0,170	0,000	
	hsCRP	0,122	0,014	0,230	0,028	
	prepregnancy	-0,060	-0,168	0,049	0,278	
	Gestational	0,186	0,079	0,293	0,001	
Free cholesterol in small HDL particles	GlycA	0,448	0,317	0,578	0,000	0,186
	Triglycerides	-0,146	-0,273	-0,019	0,025	
	hsCRP	0,108	0,003	0,212	0,044	
	prepregnancy	0,001	-0,103	0,106	0,979	
	Gestational	0,097	-0,007	0,200	0,068	
Triglycerides in chylomicrons and extremely large VLDL particles	GlycA	0,192	0,092	0,291	0,000	0,565
	Triglycerides	0,715	0,612	0,818	0,000	
	hsCRP	-0,069	-0,147	0,010	0,087	
	prepregnancy	0,018	-0,061	0,097	0,647	
	Gestational	-0,214	-0,292	-0,136	0,000	
Triglycerides in very large VLDL particles	GlycA	0,058	-0,015	0,130	0,120	0,781
	Triglycerides	1,007	0,928	1,086	0,000	
	hsCRP	-0,016	-0,073	0,042	0,591	
	prepregnancy	0,026	-0,032	0,084	0,384	
	Gestational	-0,199	-0,256	-0,142	0,000	
Triglycerides in large VLDL particles	GlycA	0,070	-0,018	0,159	0,120	0,781
	Triglycerides	1,230	1,134	1,326	0,000	
	hsCRP	-0,019	-0,090	0,051	0,591	
	prepregnancy	0,032	-0,040	0,103	0,384	
	Gestational	-0,243	-0,313	-0,173	0,000	
Triglycerides in medium VLDL	GlycA	0,052	0,000	0,104	0,050	0,870
	Triglycerides	0,934	0,884	0,985	0,000	

particles	hsCRP	-0,033	-0,075	0,009	0,119	
	prepregnancy	0,024	-0,018	0,066	0,265	
	Gestational	-0,143	-0,185	-0,102	0,000	
Triglycerides in small VLDL particles	GlycA	0,059	0,017	0,101	0,007	0,915
	Triglycerides	0,933	0,891	0,974	0,000	
	hsCRP	-0,028	-0,062	0,006	0,110	
	prepregnancy	0,015	-0,018	0,049	0,371	
	Gestational	-0,040	-0,073	-0,006	0,020	
Triglycerides in very small VLDL particles	GlycA	-0,075	-0,125	-0,025	0,004	0,880
	Triglycerides	0,945	0,896	0,994	0,000	
	hsCRP	0,009	-0,031	0,050	0,646	
	prepregnancy	0,014	-0,026	0,055	0,485	
	Gestational	0,100	0,060	0,140	0,000	
Triglycerides in IDL particles	GlycA	-0,179	-0,258	-0,099	0,000	0,697
	Triglycerides	0,839	0,762	0,917	0,000	
	hsCRP	0,035	-0,029	0,099	0,280	
	prepregnancy	0,009	-0,055	0,072	0,791	
	Gestational	0,220	0,156	0,283	0,000	
Triglycerides in large LDL particles	GlycA	-0,172	-0,262	-0,083	0,000	0,618
	Triglycerides	0,762	0,675	0,849	0,000	
	hsCRP	0,042	-0,030	0,114	0,249	
	prepregnancy	-0,013	-0,085	0,058	0,713	
	Gestational	0,260	0,189	0,331	0,000	
Triglycerides in medium LDL particles	GlycA	-0,174	-0,267	-0,082	0,000	0,591
	Triglycerides	0,746	0,656	0,836	0,000	
	hsCRP	0,039	-0,035	0,113	0,300	
	prepregnancy	-0,020	-0,094	0,055	0,604	
	Gestational	0,260	0,186	0,333	0,000	
Triglycerides in small LDL particles	GlycA	-0,096	-0,169	-0,022	0,011	0,745
	Triglycerides	0,845	0,774	0,916	0,000	
	hsCRP	0,018	-0,040	0,077	0,540	
	prepregnancy	-0,031	-0,090	0,027	0,292	
	Gestational	0,190	0,132	0,248	0,000	
Triglycerides in very large HDL particles	GlycA	-0,093	-0,213	0,028	0,131	0,307
	Triglycerides	0,528	0,410	0,645	0,000	
	hsCRP	0,017	-0,079	0,114	0,726	
	prepregnancy	-0,136	-0,233	-0,039	0,006	
	Gestational	0,182	0,086	0,278	0,000	
Triglycerides in large HDL particles	GlycA	-0,066	-0,194	0,062	0,309	0,217
	Triglycerides	0,364	0,239	0,489	0,000	
	hsCRP	0,078	-0,025	0,181	0,136	
	prepregnancy	-0,145	-0,247	-0,042	0,006	
	Gestational	0,229	0,127	0,330	0,000	
Triglycerides in medium HDL particles	GlycA	0,264	0,191	0,336	0,000	0,750
	Triglycerides	0,664	0,594	0,734	0,000	
	hsCRP	0,022	-0,036	0,080	0,459	
	prepregnancy	0,053	-0,005	0,111	0,074	
	Gestational	-0,013	-0,071	0,044	0,647	
Triglycerides in small HDL particles	GlycA	0,029	-0,024	0,082	0,283	0,864
	Triglycerides	0,890	0,838	0,942	0,000	
	hsCRP	0,013	-0,030	0,056	0,548	
	prepregnancy	0,076	0,033	0,119	0,001	
	Gestational	0,012	-0,030	0,054	0,579	
Mean diameter for VLDL particles	GlycA	0,104	0,020	0,188	0,015	0,665
	Triglycerides	0,789	0,707	0,870	0,000	
	hsCRP	-0,030	-0,097	0,037	0,377	
	prepregnancy	0,026	-0,041	0,094	0,440	
	Gestational	-0,229	-0,295	-0,162	0,000	
Mean diameter for LDL particles	GlycA	-0,240	-0,383	-0,098	0,001	0,030
	Triglycerides	0,170	0,032	0,309	0,016	
	hsCRP	0,068	-0,047	0,182	0,245	
	prepregnancy	0,101	-0,013	0,216	0,083	
	Gestational	-0,056	-0,169	0,057	0,332	

Mean diameter for HDL particles	GlycA	-0,215	-0,351	-0,078	0,002	0,115
	Triglycerides	-0,053	-0,186	0,080	0,434	
	hsCRP	0,072	-0,037	0,181	0,196	
	prepregnancy	-0,176	-0,286	-0,067	0,002	
	Gestational	0,196	0,088	0,304	0,000	
Serum total cholesterol	GlycA	0,108	-0,016	0,233	0,089	0,259
	Triglycerides	0,336	0,214	0,457	0,000	
	hsCRP	-0,047	-0,146	0,053	0,359	
	prepregnancy	-0,079	-0,179	0,021	0,119	
	Gestational	0,232	0,133	0,331	0,000	
Total cholesterol in VLDL	GlycA	0,140	0,082	0,198	0,000	0,840
	Triglycerides	0,822	0,766	0,879	0,000	
	hsCRP	-0,076	-0,122	-0,029	0,001	
	prepregnancy	-0,003	-0,049	0,044	0,913	
	Gestational	0,048	0,002	0,094	0,041	
Remnant cholesterol (non-HDL, non-LDL - cholesterol)	GlycA	0,112	0,021	0,204	0,016	0,603
	Triglycerides	0,678	0,590	0,767	0,000	
	hsCRP	-0,087	-0,160	-0,014	0,020	
	prepregnancy	-0,018	-0,091	0,055	0,625	
	Gestational	0,117	0,045	0,190	0,002	
Total cholesterol in LDL	GlycA	0,086	-0,042	0,213	0,187	0,222
	Triglycerides	0,353	0,229	0,477	0,000	
	hsCRP	-0,105	-0,207	-0,002	0,045	
	prepregnancy	-0,041	-0,143	0,062	0,436	
	Gestational	0,180	0,079	0,281	0,001	
Total cholesterol in HDL	GlycA	0,066	-0,070	0,202	0,341	0,116
	Triglycerides	-0,256	-0,389	-0,124	0,000	
	hsCRP	0,102	-0,007	0,211	0,066	
	prepregnancy	-0,145	-0,254	-0,036	0,009	
	Gestational	0,276	0,168	0,384	0,000	
Total cholesterol in HDL2	GlycA	0,067	-0,068	0,202	0,331	0,129
	Triglycerides	-0,311	-0,443	-0,180	0,000	
	hsCRP	0,103	-0,006	0,211	0,063	
	prepregnancy	-0,147	-0,256	-0,039	0,008	
	Gestational	0,261	0,154	0,369	0,000	
Total cholesterol in HDL3	GlycA	0,037	-0,087	0,160	0,562	0,268
	Triglycerides	0,267	0,146	0,388	0,000	
	hsCRP	0,078	-0,021	0,177	0,122	
	prepregnancy	-0,097	-0,197	0,002	0,055	
	Gestational	0,336	0,237	0,434	0,000	
Esterified cholesterol	GlycA	0,122	-0,004	0,247	0,057	0,249
	Triglycerides	0,310	0,188	0,432	0,000	
	hsCRP	-0,047	-0,147	0,054	0,362	
	prepregnancy	-0,082	-0,182	0,019	0,112	
	Gestational	0,237	0,138	0,337	0,000	
Free cholesterol	GlycA	0,072	-0,050	0,195	0,248	0,282
	Triglycerides	0,397	0,277	0,516	0,000	
	hsCRP	-0,046	-0,144	0,052	0,360	
	prepregnancy	-0,074	-0,172	0,024	0,140	
	Gestational	0,217	0,120	0,315	0,000	
Triglycerides in VLDL	GlycA	0,034	-0,003	0,071	0,068	0,935
	Triglycerides	0,971	0,936	1,007	0,000	
	hsCRP	-0,021	-0,050	0,009	0,163	
	prepregnancy	0,020	-0,010	0,049	0,187	
	Gestational	-0,101	-0,130	-0,072	0,000	
Triglycerides in LDL	GlycA	-0,161	-0,247	-0,074	0,000	0,643
	Triglycerides	0,777	0,693	0,861	0,000	
	hsCRP	0,042	-0,028	0,111	0,238	
	prepregnancy	-0,019	-0,088	0,051	0,596	
	Gestational	0,255	0,186	0,324	0,000	
Triglycerides in HDL	GlycA	0,029	-0,055	0,113	0,494	0,666
	Triglycerides	0,739	0,658	0,821	0,000	
	hsCRP	0,051	-0,016	0,118	0,139	

	prepregnancy	-0,061	-0,128	0,006	0,074	
	Gestational	0,148	0,082	0,215	0,000	
Total phosphoglycerides	GlycA	0,047	-0,071	0,164	0,436	0,340
	Triglycerides	0,419	0,305	0,534	0,000	
	hsCRP	0,030	-0,064	0,125	0,527	
	prepregnancy	-0,098	-0,192	-0,003	0,043	
	Gestational	0,265	0,171	0,358	0,000	
Ratio of triglycerides to phosphoglycerides	GlycA	0,155	0,106	0,204	0,000	0,884
	Triglycerides	0,869	0,821	0,916	0,000	
	hsCRP	-0,045	-0,084	-0,006	0,026	
	prepregnancy	0,017	-0,022	0,057	0,388	
	Gestational	-0,104	-0,143	-0,065	0,000	
Phosphatidylcholine and other cholines	GlycA	0,028	-0,089	0,146	0,635	0,341
	Triglycerides	0,414	0,299	0,528	0,000	
	hsCRP	0,029	-0,065	0,123	0,542	
	prepregnancy	-0,099	-0,193	-0,005	0,040	
	Gestational	0,288	0,195	0,381	0,000	
Sphingomyelins	GlycA	0,148	0,015	0,280	0,029	0,162
	Triglycerides	0,175	0,046	0,304	0,008	
	hsCRP	-0,021	-0,127	0,085	0,696	
	prepregnancy	-0,077	-0,184	0,029	0,153	
	Gestational	0,233	0,128	0,338	0,000	
Total cholines	GlycA	0,031	-0,089	0,152	0,607	0,307
	Triglycerides	0,376	0,259	0,493	0,000	
	hsCRP	0,027	-0,069	0,124	0,582	
	prepregnancy	-0,098	-0,195	-0,001	0,047	
	Gestational	0,290	0,195	0,386	0,000	
Apolipoprotein A-I	GlycA	0,155	0,019	0,292	0,026	0,108
	Triglycerides	-0,090	-0,223	0,044	0,187	
	hsCRP	0,063	-0,047	0,172	0,260	
	prepregnancy	-0,152	-0,262	-0,042	0,007	
	Gestational	0,272	0,164	0,381	0,000	
Apolipoprotein B	GlycA	0,097	0,007	0,187	0,035	0,614
	Triglycerides	0,703	0,615	0,791	0,000	
	hsCRP	-0,097	-0,169	-0,025	0,008	
	prepregnancy	-0,019	-0,091	0,053	0,606	
	Gestational	0,104	0,033	0,176	0,004	
Ratio of apolipoprotein B to apolipoprotein A-I	GlycA	0,023	-0,068	0,114	0,622	0,604
	Triglycerides	0,781	0,692	0,870	0,000	
	hsCRP	-0,139	-0,211	-0,066	0,000	
	prepregnancy	0,074	0,001	0,147	0,048	
	Gestational	-0,049	-0,122	0,023	0,179	
Total fatty acids	GlycA	0,116	0,026	0,207	0,012	0,609
	Triglycerides	0,643	0,555	0,732	0,000	
	hsCRP	-0,012	-0,085	0,060	0,739	
	prepregnancy	-0,053	-0,126	0,020	0,153	
	Gestational	0,178	0,105	0,251	0,000	
Estimated degree of unsaturation	GlycA	0,134	0,008	0,260	0,038	0,239
	Triglycerides	-0,541	-0,664	-0,418	0,000	
	hsCRP	-0,089	-0,190	0,013	0,086	
	prepregnancy	-0,071	-0,173	0,030	0,167	
	Gestational	0,018	-0,084	0,119	0,732	
22:6, docosahexaenoic acid	GlycA	0,155	0,034	0,276	0,012	0,303
	Triglycerides	0,408	0,290	0,526	0,000	
	hsCRP	-0,031	-0,128	0,066	0,530	
	prepregnancy	-0,054	-0,151	0,043	0,274	
	Gestational	0,134	0,037	0,231	0,007	
18:2, linoleic acid	GlycA	0,147	0,029	0,265	0,014	0,340
	Triglycerides	0,367	0,252	0,482	0,000	
	hsCRP	-0,023	-0,117	0,072	0,636	
	prepregnancy	-0,108	-0,202	-0,013	0,025	
	Gestational	0,257	0,162	0,351	0,000	
Omega-3 fatty acids	GlycA	0,184	0,069	0,299	0,002	0,371

	Triglycerides	0,446	0,334	0,558	0,000	
	hsCRP	-0,039	-0,131	0,053	0,400	
	prepregnancy	-0,071	-0,163	0,021	0,132	
	Gestational	0,137	0,045	0,230	0,004	
Omega-6 fatty acids	GlycA	0,166	0,049	0,283	0,005	0,349
	Triglycerides	0,376	0,262	0,490	0,000	
	hsCRP	-0,025	-0,118	0,069	0,606	
	prepregnancy	-0,092	-0,185	0,002	0,055	
	Gestational	0,238	0,144	0,331	0,000	
Polyunsaturated fatty acids	GlycA	0,174	0,059	0,289	0,003	0,374
	Triglycerides	0,401	0,290	0,513	0,000	
	hsCRP	-0,028	-0,119	0,064	0,554	
	prepregnancy	-0,091	-0,182	0,001	0,053	
	Gestational	0,226	0,134	0,318	0,000	
Monounsaturated fatty acids; 16:1, 18:1	GlycA	0,095	0,030	0,160	0,004	0,799
	Triglycerides	0,782	0,718	0,845	0,000	
	hsCRP	0,002	-0,050	0,054	0,937	
	prepregnancy	-0,011	-0,063	0,042	0,690	
	Gestational	0,140	0,088	0,192	0,000	
Saturated fatty acids	GlycA	0,069	-0,021	0,160	0,133	0,609
	Triglycerides	0,690	0,601	0,778	0,000	
	hsCRP	-0,010	-0,082	0,063	0,787	
	prepregnancy	-0,050	-0,123	0,023	0,178	
	Gestational	0,147	0,075	0,220	0,000	
Ratio of 22:6 docosahexaenoic acid to total fatty acids	GlycA	0,146	0,001	0,291	0,049	-0,001
	Triglycerides	-0,091	-0,232	0,051	0,207	
	hsCRP	-0,060	-0,176	0,056	0,309	
	prepregnancy	-0,025	-0,141	0,091	0,672	
	Gestational	0,021	-0,095	0,138	0,718	
Ratio of 18:2 linoleic acid to total fatty acids	GlycA	0,053	-0,055	0,161	0,335	0,447
	Triglycerides	-0,701	-0,807	-0,596	0,000	
	hsCRP	-0,030	-0,116	0,057	0,500	
	prepregnancy	-0,095	-0,182	-0,009	0,030	
	Gestational	0,123	0,037	0,209	0,005	
Ratio of omega-3 fatty acids to total fatty acids	GlycA	0,213	0,071	0,355	0,003	0,039
	Triglycerides	0,030	-0,108	0,169	0,665	
	hsCRP	-0,076	-0,190	0,038	0,190	
	prepregnancy	-0,070	-0,184	0,044	0,225	
	Gestational	0,040	-0,074	0,154	0,490	
Ratio of omega-6 fatty acids to total fatty acids	GlycA	0,094	0,016	0,173	0,019	0,707
	Triglycerides	-0,896	-0,972	-0,819	0,000	
	hsCRP	-0,041	-0,104	0,022	0,197	
	prepregnancy	-0,054	-0,117	0,009	0,091	
	Gestational	0,065	0,002	0,127	0,045	
Ratio of polyunsaturated fatty acids to total fatty acids	GlycA	0,158	0,072	0,244	0,000	0,650
	Triglycerides	-0,889	-0,972	-0,805	0,000	
	hsCRP	-0,066	-0,134	0,003	0,061	
	prepregnancy	-0,075	-0,144	-0,006	0,033	
	Gestational	0,078	0,009	0,146	0,027	
Ratio of monounsaturated fatty acids to total fatty acids	GlycA	-0,020	-0,093	0,054	0,600	0,742
	Triglycerides	0,839	0,768	0,911	0,000	
	hsCRP	0,060	0,001	0,119	0,046	
	prepregnancy	0,091	0,032	0,150	0,003	
	Gestational	0,003	-0,056	0,062	0,921	
Ratio of saturated fatty acids to total fatty acids	GlycA	-0,315	-0,451	-0,179	0,000	0,125
	Triglycerides	0,467	0,335	0,600	0,000	
	hsCRP	0,037	-0,071	0,146	0,497	
	prepregnancy	0,004	-0,105	0,113	0,940	
	Gestational	-0,172	-0,280	-0,063	0,002	
Glucose	GlycA	0,384	0,246	0,521	0,000	0,100
	Triglycerides	-0,182	-0,315	-0,048	0,008	
	hsCRP	-0,054	-0,164	0,056	0,335	
	prepregnancy	0,061	-0,050	0,171	0,280	

	Gestational	-0,147	-0,256	-0,038	0,008	
Lactate	GlycA	0,210	0,068	0,351	0,004	0,047
	Triglycerides	0,057	-0,081	0,195	0,417	
	hsCRP	-0,037	-0,150	0,076	0,521	
	prepregnancy	0,016	-0,098	0,129	0,787	
	Gestational	0,009	-0,103	0,122	0,869	
Pyruvate	GlycA	0,262	0,125	0,399	0,000	0,102
	Triglycerides	0,067	-0,067	0,200	0,327	
	hsCRP	-0,017	-0,127	0,093	0,759	
	prepregnancy	0,061	-0,049	0,171	0,274	
	Gestational	0,052	-0,057	0,161	0,350	
Citrate	GlycA	-0,192	-0,334	-0,049	0,009	0,032
	Triglycerides	0,209	0,070	0,348	0,003	
	hsCRP	0,046	-0,068	0,160	0,424	
	prepregnancy	0,128	0,013	0,242	0,029	
	Gestational	-0,044	-0,157	0,069	0,441	
Glycerol	GlycA	0,269	0,131	0,407	0,000	0,149
	Triglycerides	-0,022	-0,157	0,113	0,749	
	hsCRP	0,015	-0,092	0,123	0,777	
	prepregnancy	0,245	0,137	0,352	0,000	
	Gestational	-0,054	-0,160	0,053	0,321	
Alanine	GlycA	0,349	0,219	0,480	0,000	0,187
	Triglycerides	0,155	0,027	0,282	0,017	
	hsCRP	-0,042	-0,146	0,063	0,433	
	prepregnancy	-0,007	-0,111	0,098	0,901	
	Gestational	-0,013	-0,116	0,091	0,810	
Glutamine	GlycA	0,212	0,071	0,354	0,003	0,045
	Triglycerides	-0,277	-0,415	-0,139	0,000	
	hsCRP	-0,026	-0,140	0,087	0,648	
	prepregnancy	-0,099	-0,212	0,015	0,087	
	Gestational	-0,028	-0,141	0,084	0,619	
Glycine	GlycA	0,179	0,039	0,320	0,013	0,056
	Triglycerides	-0,200	-0,337	-0,063	0,004	
	hsCRP	-0,107	-0,220	0,006	0,062	
	prepregnancy	-0,030	-0,143	0,083	0,602	
	Gestational	-0,156	-0,267	-0,044	0,006	
Histidine	GlycA	0,073	-0,071	0,217	0,320	0,013
	Triglycerides	0,029	-0,111	0,169	0,681	
	hsCRP	-0,024	-0,139	0,092	0,688	
	prepregnancy	-0,074	-0,189	0,041	0,207	
	Gestational	0,104	-0,010	0,219	0,073	
Isoleucine	GlycA	0,326	0,219	0,433	0,000	0,455
	Triglycerides	0,459	0,355	0,564	0,000	
	hsCRP	-0,017	-0,102	0,069	0,703	
	prepregnancy	0,023	-0,063	0,109	0,595	
	Gestational	-0,192	-0,277	-0,108	0,000	
Leucine	GlycA	0,372	0,249	0,494	0,000	0,283
	Triglycerides	0,223	0,103	0,342	0,000	
	hsCRP	0,020	-0,078	0,119	0,682	
	prepregnancy	0,048	-0,051	0,146	0,340	
	Gestational	-0,180	-0,277	-0,083	0,000	
Valine	GlycA	0,191	0,054	0,329	0,006	0,101
	Triglycerides	-0,084	-0,218	0,050	0,219	
	hsCRP	-0,001	-0,111	0,108	0,979	
	prepregnancy	0,148	0,038	0,258	0,008	
	Gestational	-0,243	-0,352	-0,134	0,000	
Phenylalanine	GlycA	0,224	0,092	0,356	0,001	0,170
	Triglycerides	0,088	-0,040	0,217	0,179	
	hsCRP	0,084	-0,022	0,189	0,121	
	prepregnancy	0,155	0,049	0,261	0,004	
	Gestational	0,098	-0,007	0,202	0,067	
Tyrosine	GlycA	0,113	-0,030	0,255	0,122	0,023
	Triglycerides	-0,066	-0,205	0,074	0,355	

	hsCRP	0,039	-0,075	0,154	0,499	
	prepregnancy	0,023	-0,091	0,138	0,688	
	Gestational	-0,164	-0,278	-0,051	0,005	
Acetate	GlycA	-0,164	-0,308	-0,021	0,025	0,017
	Triglycerides	0,066	-0,074	0,205	0,357	
	hsCRP	-0,057	-0,172	0,058	0,330	
	prepregnancy	9,837E-05	-0,115	0,115	0,999	
	Gestational	0,104	-0,010	0,217	0,075	
Acetoacetate	GlycA	-0,158	-0,299	-0,017	0,029	0,048
	Triglycerides	0,159	0,022	0,297	0,024	
	hsCRP	0,049	-0,064	0,162	0,398	
	prepregnancy	0,158	0,044	0,271	0,007	
	Gestational	-0,143	-0,255	-0,031	0,013	
3-hydroxybutyrate	GlycA	0,179	0,037	0,321	0,014	0,034
	Triglycerides	-0,124	-0,263	0,014	0,079	
	hsCRP	-0,042	-0,156	0,072	0,466	
	prepregnancy	0,157	0,043	0,271	0,007	
	Gestational	0,001	-0,112	0,113	0,993	
Creatinine	GlycA	0,025	-0,120	0,169	0,737	0,008
	Triglycerides	-0,035	-0,175	0,106	0,628	
	hsCRP	0,074	-0,042	0,189	0,211	
	prepregnancy	-0,025	-0,140	0,091	0,674	
	Gestational	-0,134	-0,249	-0,020	0,022	
Albumin	GlycA	0,640	0,517	0,764	0,000	0,270
	Triglycerides	-0,424	-0,544	-0,303	0,000	
	hsCRP	-0,118	-0,217	-0,019	0,020	
	prepregnancy	-0,093	-0,192	0,007	0,068	
	Gestational	-0,233	-0,331	-0,135	0,000	
